# Poptosis or Peptide-Induced Transmembrane Pore Formation: A Novel Way to Kill Cancer Cells without Affecting Normal Cells †

**DOI:** 10.3390/biomedicines12061144

**Published:** 2024-05-22

**Authors:** Matthew R. Pincus, Miriam Silberstein, Nitzan Zohar, Ehsan Sarafraz-Yazdi, Wilbur B. Bowne

**Affiliations:** 1 Department of Pathology, SUNY Downstate Medical Center, 450 Clarkson Avenue, Brooklyn, NY 11203, USA; 2 Touro College, 1602 Avenue J, Brooklyn, NY 10036, USA; msilbers3@student.touro.edu; 3 Department of Surgery, Jefferson Pancreas, Biliary and Related Cancer, Thomas Jefferson University, Philadelphia, PA 19107, USA; nitzan.zohar@jefferson.edu (N.Z.); wilbur.bowne@jefferson.edu (W.B.B.); 4 NomoCan, 310 East 67th Street, New York, NY 10065, USA; ehsan.yazdi@nomocan.com

**Keywords:** PNC-27, tumor cell necrosis, transmembrane pore formation, PNC-27–HDM-2 membrane complexes

## Abstract

Recent advances in cancer treatment like personalized chemotherapy and immunotherapy are aimed at tumors that meet certain specifications. In this review, we describe a new approach to general cancer treatment, termed peptide-induced poptosis, in which specific peptides, e.g., PNC-27 and its shorter analogue, PNC-28, that contain the segment of the p53 transactivating 12–26 domain that bind to HDM-2 in its 1–109 domain, bind to HDM-2 in the membranes of cancer cells, resulting in transmembrane pore formation and the rapid extrusion of cancer cell contents, i.e., tumor cell necrosis. These peptides cause tumor cell necrosis of a wide variety of solid tissue and hematopoietic tumors but have no effect on the viability and growth of normal cells since they express at most low levels of membrane-bound HDM-2. They have been found to successfully treat a highly metastatic pancreatic tumor as well as stem-cell-enriched human acute myelogenous leukemias in nude mice, with no evidence of off-target effects. These peptides also are cytotoxic to chemotherapy-resistant cancers and to primary tumors. We performed high-resolution scanning immuno-electron microscopy and visualized the pores in cancer cells induced by PNC-27. This peptide forms 1:1 complexes with HDM-2 in a temperature-independent step, followed by dimerization of these complexes to form transmembrane channels in a highly temperature-dependent step parallel to the mode of action of other membranolytic but less specific agents like streptolysin. These peptides therefore may be effective as general anti-cancer agents.

## 1. Introduction

Chemotherapy, especially together with immunotherapy, has resulted in major advances in cancer treatment [1,2]. The development of personalized chemotherapy using inhibitors of specific signal transduction proteins such as specific forms of oncogenic ras-p21 [3] have likewise resulted in improved cancer treatment modalities, especially in relation to solid tissue tumors such as colorectal cancer [4] and lung cancer [5]. These recently developed methods still have off-target effects that include the development of autoimmune states in patients treated with anti-PD1 (programmed cell death protein on the surface of T cells) and CTLA (cytotoxic T-lymphocyte-associated protein 4) agents [6] and sometimes compromised efficacy when tumors undergo changes such as mutations or use of alternate signal transduction pathways [1].

This review concerns the recent discovery of a set of peptides, the main prototype of which is PNC-27, containing p53 residues 12–26, from its transactivating HDM-2-protein-binding domain, attached to a transmembrane-penetrating peptide sequence, described below, that kills a wide variety of cancer cells, including solid tissue tumors and hematopoietic cancers, but that has no effects on the viability and growth of normal cells. The mechanism involves the interaction of these peptides with HDM-2 (human-double-minute-binding protein) expressed in the cell membranes of cancer cells but expressed either at low levels of or not at all in the membranes of normal cells. Colocalization of these peptides with membrane-expressed HDM-2 results in the formation of transmembrane pores, leading to the explosive release of the cancer cells’ intracellular contents, resulting in tumor cell necrosis, termed poptosis. These peptides have been tested in vivo against human tumors and have been found to eradicate them. The advantage in the use of these peptides is that their anti-cancer effects do not depend on the inhibition of intracellular mechanisms such as specific signal transduction pathways, specific proteins on these pathways, specific DNA-repair processes, DNA methylation, or the inhibition of multiple drug-resistant (MDR) gene products, giving them the potential to treat many different forms of cancer with the only requirement being that they interact with cancer-cell-membrane-expressed HDM-2.

It should be noted that several other peptides have been developed that have also been found to induce pore formation in cancer cells. Several membrane-active antibiotic peptides such as magainin, which contains positively charged amino acid residues, allowing for an interaction with the negatively charged surfaces of cells, have been found to be membranolytic, preferentially to cancer cells [7], but can also affect normal cells. Certain cell-penetrating peptides (CPP), that enable the delivery of cargo, such as peptides and poly-nucleic acids into cells, target cancer cells that have been found to contain significant levels of gangliosides in their membranes that are not present in normal cells [8]. Other peptides have been found to bind preferentially to cancer cell membranes. An example of the use of cancer-cell-targeting CPPs is the use of the peptide derived from the antimicrobial peptide buforin, called BR2 (whose sequence is RAGLQFPVGRLLRRLLR). This peptide was found to enter cancer cells selectively (it did enter normal cells, although to a significantly less degree) and was used to deliver the inactivating anti-ras-p21 antibody (Y13-259) into cancer cells, resulting in tumor cell necrosis. This effect was abolished if the cells were incubated with a ganglioside synthesis inhibitor, strongly suggesting that the ganglioside composition of cancer cells may be different from that of untransformed cells, allowing them to be targeted by agents that bind to membrane-expressed gangliosides [8].

Using this approach, a series of decoy peptides were designed to block the activation of the AAC-11 anti-apoptotic protein in tumor cells, most notably in Sezary’s leukemia cells [9,10,11]. AAC-11 is thought to activate PAK1 (p21-activated kinase) on its anti-apoptotic pathway. These peptides contained the heptad leucine zipper sequence involved in the activation domain of AAC-11 and were attached on the amino termini to a CPP (penetratin) sequence. These peptides were found to colocalize with PAK1 in the cell membranes of Sezary’s leukemia cells in vitro and in vivo, and they induced cell death via pore formation and apoptosis. The release of damage-associated molecular pattern agents (DAMPs) results in the activation of immune responses to the cancer cells. Thus, besides inducing tumor cell killing, these peptides appear to activate an anti-tumor immune response. These peptides have yet to be tested against other human cancers [11].

In this current review, we show that specific peptides from the HDM-2-binding domain of p53 attached to a CPP from *antennapaedia* on their carboxyl terminal ends induce tumor cell necrosis of a wide range of cancers, including solid tissue and hematopoietic cancers, by inducing transmembrane pores, and that they have no effect on the growth and viability of a wide variety of normal cells. These peptides act by binding to the HDM-2 protein, which is expressed significantly in the membranes of cancer but not normal cells. When bound to membrane-attached HDM-2, the peptide-HDM-2 complexes form transmembrane pores that can be directly observed in high-resolution immuno-scanning electron microscopy.

## 2. Design and Effects of PNC-27 and PNC-28

The most effective of these poptosis-inducing agents is PNC-27, which contains amino acid residues 12–26 of the transactivating domain of p53, which have been found to be involved in the binding of p53 to the H(M)DM-2 protein [12]. This sequence is attached to a transmembrane-penetrating sequence, also called penetratin, as shown in entry 1 of Table 1 [13]. The leader sequence was added to this peptide (and other peptides used in our studies, such as those shown in Table 1) to induce the crossing of the peptides across the cell membrane since the isolated p53 12–26 peptide had no effect on cancer cells due to its inability to cross the cell membrane [13].

The second peptide shown in Table 1 is PNC-28, which is a shorter version of PNC-27 and contains p53 residues 17–26 that contain the p53 residues that contacts HDM-2, and has anti-cancer activity similar to that of PNC-27. The third peptide shown in Table 1 is a negative control peptide, containing a sequence (×13) from human cytochrome p450 attached to the leader sequence on its carboxyl terminal end. H (human) or M (mouse) DM2 (double minute-binding protein-2) is mainly a nuclear protein that is an E3 ubiquitin ligase that binds to p53 in its amino terminal domain (residues 1–109) and several other proteins that target these proteins for ubiquitination and degradation in the proteosome [12]. Thus, HDM-2 limits the ability of p53 to stimulate the apoptosis of transformed cells.

PNC-27 was originally designed by our research group to block the binding of p53 to HDM-2 in the nuclei of cancer cells as a “decoy” peptide, i.e., the peptide would cross the cancer cell and nuclear membranes using its penetratin sequence and bind to HDM-2 in place of the full p53 protein, hopefully enhancing p53-induced apoptosis of the cancer cells [13].

### 2.1. PNC-27 and 28 Induce Tumor Cell Necrosis of Multiple Different Types of Cancers

**Figure 1:**
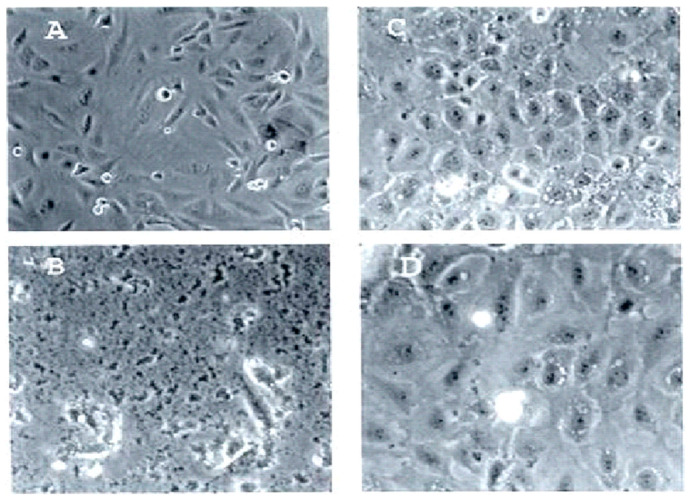
Effects of PNC-28 on normal rat pancreatic acinar cells (BMRPA1 cells) and rat pancreatic acinar cancer cells (TUC-3 cells). Approximately 20,000 cells were incubated with PNC-28 (100 μg/mL) for 72 h. (**A**) TUC-3 cells prior to treatment with PNC-28, showing malignant spindle-shaped cells. (**B**) TUC-3 cells after treatment with PNC-28, showing no viable cells and only cellular debris. (**C**) Normal counterpart rat pancreatic BMRPA1 cells showing well-differentiated round acinar cells. (**D**) BMRPA1 cells after treatment with PNC-28, showing normal, rounded acinar cells. There was no cell death. Adapted from ref. [13] with permission by Bentham Science.

In multiple studies, we found that PNC-27 and PNC-28 (Table 1) induced rapid cancer cell necrosis but did not affect untransformed cells [13], as illustrated in Figure 1, wherein rat pancreatic acinar cancer cells called TUC-3 (the spindle-shaped cancer cells shown in Figure 1A) and their normal counterpart pancreatic acinar cells called BMRPA1 (shown in Figure 1B) were incubated with PNC-28 for 24 h. As shown in Figure 1C, all the TUC-3 cells were killed by PNC-28, while, as shown in Figure 1D, the BMRPA1 cells remained viable [13].

In further studies, PNC-27 and PNC-28 have been found to be cytotoxic to a large variety of human and mammalian solid tissue and non-solid tissue cancer cells, including pancreatic [13], breast [13], ovarian [14], colon [15], cervical (HeLa) [13], non-small cell lung carcinoma [13], angiosarcoma [13], osteogenic sarcoma [13], acute myelogenous leukemia [16,17], and chronic myelogenous leukemia [18], with IC_50_ values that ranged from 6–80 μM, while neither peptide was found to be cytotoxic to untransformed control cells that included human fibroblasts [13], pancreatic acinar cells [13], human breast epithelial cells [13], keratinocytes [13], human umbilical vein (HUVEC) cells [14], and rat mononuclear cells [16] at the highest concentrations used with the corresponding cancer cells. Importantly, PNC-28 was found to have no effect on the abilities of human hematopoietic stem cells from the cord blood of five different donors to differentiate into mature hematopoietic cells in the presence of growth factors [13], strongly suggesting that neither peptide would suppress bone marrow during cancer treatment. This finding was further confirmed in a recent study [17], showing that PNC-27 was cytotoxic to human AML stem cells in vitro and in vivo but had no effect on normal human myeloid stem cells.

Typical dose–response curves for the cancer cell cytotoxicity of PNC-27 are shown in [14,15,16] for ovarian cancers, colon cancers, and acute myelogenous leukemias. PNC-27 killed over 90 percent of these tumor cells (approximately 1 × 10^6^) in a four-hour period. On the other hand, it can be seen in these figures that the negative control PNC-29 peptide had no effect on any of these cell lines. In addition, included for each cancer cell line, a corresponding normal cell line was likewise incubated with PNC-27 and PNC-29. For SKOV-3 cells, the control cell line was HUVEC cells (umbilical vein endothelial cells) for HCT-116 CCD-18Co colonic fibroblasts, and for U-937, rat mononuclear cells. Neither PNC-27 nor PNC-29 affected these untransformed cells. Importantly, PNC-27 was also found to be cytotoxic to OVCAR-3 cells that are multi-drug-resistant [14], indicating that cancer cell killing was independent of intracellular processes.

Included among the cancer cells tested with PNC-27 were primary ovarian cancer cells, i.e., cells that were removed from patients who underwent excision of these tumors under an IRB protocol and that were then incubated in culture with PNC-27. Complete killing of these cells by 60 μM PNC-27 (by not by PNC-29) was achieved after 4 h incubation, suggesting the efficacy of this peptide in the treatment of ovarian cancer. In addition, PNC-27 was found to be cytotoxic to cancer stem cells, including colon cancer cell lines such as CTA, CTR, and SW1222 [15], that are enriched in CD44-positive tumor stem cells. As discussed below, PNC-27 is cytotoxic to human acute myelogenous tumor stem cells [17] without affecting normal hematopoietic stem cells [13,17], as discussed above. Thus, PNC-27 kills primary human tumors, chemoresistant cancers, and tumor stem cells without affecting normal cells.

### 2.2. PNC-28 Kills TUC-3 Cells In Vivo

As discussed above, PNC-27 and PNC-28 kill murine TUC-3 pancreatic cancer cells, but not their counterpart normal pancreatic acinar BMRPA1 cells, in culture. TUC-3 cells form highly metastatic tumors in nude mice within two weeks after transplantation. We implanted TUC-3 cells in nude mice either intraperitoneally or subdermally and treated these mice with continuous infusion of PNC-28 or negative control PNC-29 over a two-week period using intraperitoneally placed Alzet minipumps. PNC-28 eradicated these tumors after two weeks; no tumor growth was observed over the successive two-week period. In contrast, tumors treated with PNC-29 were found to grow to large (3500 mg) tumors that metastasized over the four-week period. In the mice treated with PNC-28, there was no evidence of undesirable side effects in that the treated mice thrived and gained weight that was statistically insignificantly different from a control group of untreated mice [13]. As discussed below, PNC-27 has been found to eradicate human-stem-cell-enriched acute myelogenous leukemia in vivo [17].

### 2.3. PNC-27 Eradicates Human Acute Myelogenous Leukemia (AML) in Nude Mice

Although most of our studies on PNC-27 and PNC-28 have been focused on solid tissue tumors, both in vitro and in vivo, we also found that these peptides are cytotoxic to hematopoietic cell cancers, including AML [16] and CML (chronic myelogenous leukemia) [18], in vitro. Recently, a large in vivo study [17] was performed at the City of Hope Medical Center in Los Angeles on stem-cell-enriched acute myelogenous leukemia (AML) cells harvested from the bone marrows from nine different patients. These cells were transplanted into the bone marrows of nude mice, which developed AML and high white cell counts. These mice were then treated with daily intraperitoneal injections of PNC-27 over a three-week period. At the end of this period, the white cell counts normalized. Bone marrow cells were then explanted from these mice into the bone marrows of naïve mice whose white cell counts were followed and found to be normal. Both cohorts of mice showed markedly prolonged Kaplan–Meier survival curves compared with those of treated mice with a negative control peptide, PNC-27.

## 3. PNC-27 and PNC-28 Are Cytotoxic to Cancer Cells by a p53-Independent Mechanism

An unexpected finding obtained for the cell lines studied was that PNC-27 and 28 both induced rapid cell death, which began to occur minutes after incubation of cell lines with either peptide but was not observed in cells undergoing apoptosis. For many tumor cell lines, complete tumor cell death occurred in 4 h. In none of these studies were the usual markers for apoptosis expressed, such as DNA laddering [13], annexin V labeling of the membrane phospholipid, and caspase expression [13,14,15,16,19]. In contrast, the release of LDH occurred almost immediately after the incubation of cell lines, with either peptide indicative of tumor cell death.

### The Leader Sequence of PNC-27 and 28 Cause the Mechanism of Cell Killing from Apoptosis to Tumor Cell Necrosis

**Figure 2:**
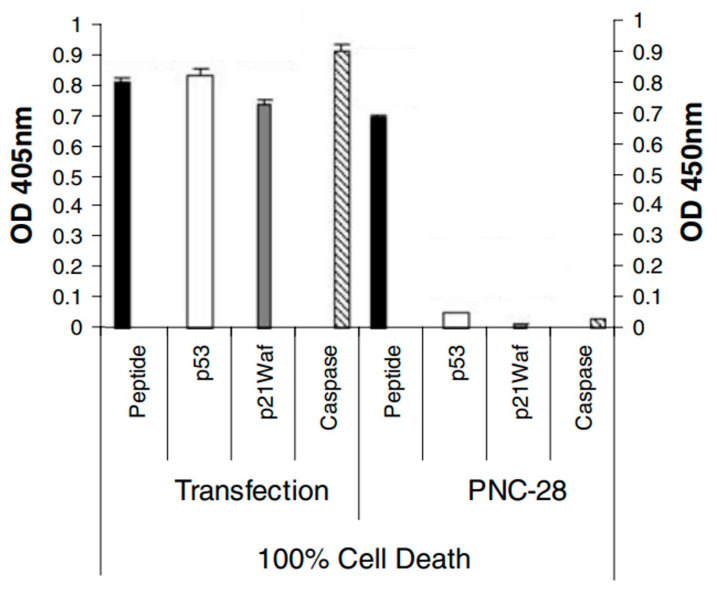
Isolated p53 17–26 peptide caused the apoptosis of MIA-PaCa-2 pancreatic cancer cells and increased intracellular levels of p53, in contrast to PNC-28 that induced tumor cell necrosis. The isolated peptide was introduced into the cells via the transfection of a plasmid expressing the 17–26 peptide intracellularly. For both treatments (75 μg/mL PNC-28 and plasmid transfection), after incubation of cells for 24 h, levels of intracellular peptide, p53, and the apoptosis marker protein p21^waf^ were measured by Western blotting, while caspase activity was measured by assay. The left ordinate shows the absorbance results for the caspase activity assay, and the right ordinate shows the band intensity for each Western blot. Actin controls showed total levels that were the same for the two sets of conditions (modified from [19]).

Since PNC-27 and 28 peptides were designed to bind to HDM-2 using the p53 12 (17)-26 sequence in the nucleus, we designed a plasmid [19] that uniquely encoded this p53 sequence and transfected it into MIA-PaCa-2 pancreatic carcinoma cells. As shown in Figure 2, these transfected cells expressed high levels of caspase-3 and p21^waf^ (another marker for apoptosis), as opposed to MIA-PaCa-2 cells that were incubated with PNC-28. In addition, p53 was found to be elevated in the transfected cells but not in the cells treated with PNC-28, as would be expected if the plasmid-expressed p53 peptide blocked HDM-2-induced degradation of the p53 protein. On the other hand, LDH release occurred almost immediately in the cells treated with PNC-28, but not in the transfected cells, pointing to a major difference in the mechanism of cancer cell killing.

Thus, the p53 peptide directly introduced into the cancer cells caused apoptosis, as was expected for the p53 peptide attached to the leader sequence. Evidently, the leader sequence of PNC-27 and -28 changed the mechanism of cancer cell death induced by both peptides, resulting in rapid tumor cell necrosis. Since the activation of p53 would be expected to result in apoptosis of the tumor cells, this novel mechanism apparently does not involve p53 activation.

This conclusion was verified by our finding that several cell lines killed by PNC-27 and PNC-28, such as MDA-MB-453 breast cancer cells [13] and SAOS2 osteogenic sarcoma cells [13], are known to have the p53 gene homozygous deleted. Thus, both peptides must be inducing cancer cell death by a non-p53-dependent mechanism, explaining the absence of the expected apoptosis.

## 4. Both PNC-27 and PNC-28 Induce Transmembrane Pore Formation, Resulting in the Extrusion of Cancer Cell Contents in a New Process Termed “Poptosis”

**Figure 3:**
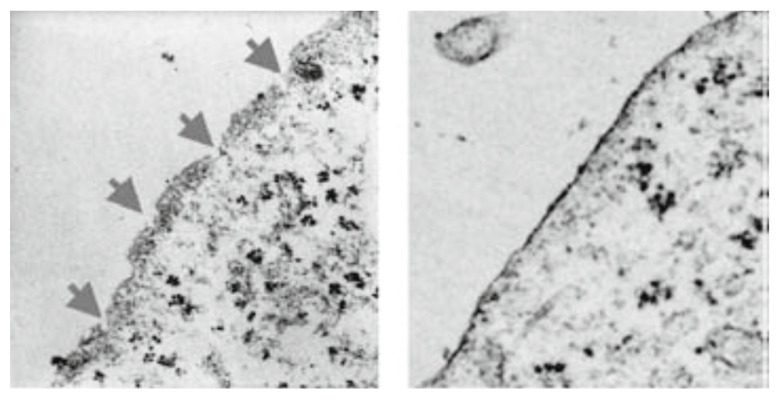
Transmission electron microscopy of untreated MIA-PaCa-2 human pancreatic cancer cells (right frame) and MIA-PaCa-2 cells treated for 15 min with 100 μg/mL PNC-28 for 15 min in PBS (left frame). The arrows in the left frame point to discrete pores formed at regular intervals in the cancer cell membrane (reprinted from [19] by permission Annals of Surgical Oncology).

Since there is a rapid release of LDH indicative of membrane damage, we subjected tumor cells, i.e., breast cancer cells (MDA-MB-468) [13], pancreatic cancer cells (MIA-PaCa-2) [13], and melanoma cells (A2058) [13], to transmission electron microscopy after incubating them for 15 min with PNC-27 or PNC-28. As illustrated in Figure 3 for MIA-PaCa-2 cells incubated with PNC-28, pores were seen to form across the cell membranes of these cells, which were not seen in the case of untreated cells. As discussed below, no pore formation was observed in the electron microscopy when untransformed cells were incubated with either peptide. Time lapse photographs of cancer cells treated with PNC-27 show that this process is explosive, hence the term “poptosis”. The question arose as to how these PNC-27 and PNC-28 induced such pores.

### PNC-27 Adopts a Membrane Active Conformation

To attempt to answer this question, we undertook to determine the three-dimensional structure of PNC-27 determined two-dimensional NMR in polar and non-polar solvents. We found that in both solvents, PNC-27 adopted an amphipathic alpha-helix-turn-alpha helix structure [13]. The two helices were formed with the amino terminal residues corresponding to p53 residues 12–26, followed by a bend at the first two residues of the leader sequence, followed by another alpha helix for the remainder of this domain. Hydrophobic residues lined one surface of this structure, while the hydrophilic residues projected from the opposite face, resulting in a so-called amphipathic conformation. Interestingly, the hydrophilic domain was sectioned into discreet blocks of negatively charged amino acid residues and positively charged amino acid residues. Amphipathic helices have been observed in several so-called membrane active peptides that induce the lysis of cell membranes, presumably by forming transmembrane pores. These include melittin [20], an active component of bee venom, which induces the lysis of erythrocytes, and magainin, an anti-microbial peptide that induces the lysis of bacterial cell walls or membranes [21].

We further found that the PNC-27 structure is superimposable on the X-ray crystal structure of a p53 peptide, corresponding to residues 15–29 bound to residues 1–109 of HDM-2, the binding site of HDM-2 for p53 [12,13]. PNC-27 has p53 residues 12–26, and we found that residues 15–26 of PNC-27 could be superimposed on the same residues of the X-ray structure [13]. The findings that PNC-27 adopts a membrane active conformation whose p53 domain structure is superimposable on that of the corresponding domain of p53 bound to HDM-2 suggested that PNC-27 may bind to HDM-2 in the cancer cell membrane.

## 5. Membrane-Bound HDM-2 Is Expressed Uniquely in Cancer Cells

Western blots of the cell membrane and nuclear fractions of a series of untransformed cells (MCF-10-2A human breast epithelial cells, BMRPA1 rat pancreatic acinar cells, and AG13145 human fibroblast cells) and another series of cancer cells (TUC-3 rat pancreatic acinar cancer cells, MIA-PaCa-2 human pancreatic cancer cells, MCF-7 human breast cancer cells, and A2058 human melanoma cancer cells) showed that all cell lines expressed significant levels of nuclear HDM-2. However, all four cancer cells lines were found to have high levels of expression of HDM-2 in their membrane fractions, while the three untransformed cell lines expressed low or no observable levels of HDM-2 in their membrane fractions [13]. Similar results have now been confirmed in a variety of different cancers including ovarian cancers (SKOV-3, OVCAR-3, HUVEC negative control) [14], colon cancers (CTA, CTP, CTR, SW1222, HCT116, CT26, CCD-18Co negative control [15]), AML (U937, OCI-AML-3, HL60, rat mononuclear cells negative control) [16], and human-stem-cell-enriched AML cells (normal stem cells negative control) [17]. Since cancer cells uniquely express HDM-2 in their cell membranes, we investigated whether PNC-27 and -28 interact with membrane-bound HDM-2.

### 5.1. PNC-27 Colocalizes with HDM-2 in the Cancer Cell Membrane

To determine whether our peptides interacted with cancer-cell-membrane-bound HDM-2, we performed co-localization studies on cancer cells incubated with PNC-27. In these studies, cancer cells were incubated for short periods with PNC-27 and then incubated with two antibody systems: one a green-fluorescent-labeled anti-PNC-27 monoclonal antibody (DO1) and the other a red-fluorescent-labeled polyclonal anti-HDM-2 antibody [13,14,15,16]. Colocalization was indicated by the presence of merged green and red fluorescence to generate yellow emission.

**Figure 4:**
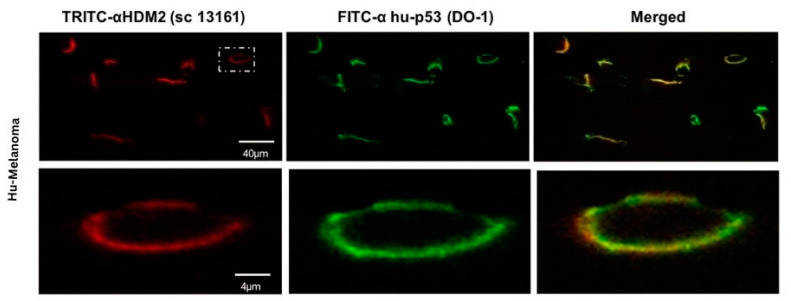
Confocal microscopic study showing that PNC-27 colocalized with HDM-2 in the membranes of A2058 human melanoma cells. A2058 cells were incubated for 15 min with 100 μg/mL of PNC-27, washed, and then incubated with two antibody systems labeled with red (HDM-2) (TRITC) fluorescent dye and green (PNC-27) (FITC) fluorescent dye. (**Upper panels**) show a group of cells showing surface red fluorescence (leftmost frame), green surface fluorescence (middle frame), and merged yellow surface fluorescence (rightmost frame), indicating colocalization of PNC-27 and HDM-2. (**Lower panels**) show magnification of a representative single cell (white dashed lines in upper leftmost frame) that shows only membrane red (leftmost panel) fluorescence, green membrane fluorescence (middle panel), and extensive merged yellow membrane fluorescence (rightmost panel). Reprinted with permission from Bentham Science [13].

An example is shown in Figure 4 for A2058 human melanoma cells. In this figure, A2058 human melanoma cells were incubated in the above-described manner with PNC-27 and the two labeled antibody systems. The top panels show low power cells in which PNC-27 labeled green (leftmost panel) and HDM-2 labeled red (middle panel) fluoresced, showing yellow fluorescence as merged images, indicating that both fluorescent probes must be proximate to one another. The two lower sets of panels were high-powered views of two such cells, showing that all the fluorescence is on the cell membrane. The combined green and red fluorescence is seen to give a yellow-colored fluorescence due to the proximity of the probes in the cell membrane to one another, indicating a colocalization of PNC-27 with HDM-2. Identical results have been obtained on numerous other cancer cell lines, including TUC-3, MIA-PaCa-2, MCF-7, SKOV-3, OVCAR-3, HCT-116, CT-26, CTA, CTP, CTR, SW1222, U937, OCI-AML3, and HL60, but not on any normal cells, including MCF-10-2A, BMRPA1, AG13145, HUVEC, CCD-18Co, and rat mononuclear cells [13,14,15,16]. Importantly, in a major in vivo study of the effect of PNC-27 on human acute myelogenous leukemia cells transplanted into the bone marrows of nude mice, the IC_50_ of PNC-27 in killing these tumors in vivo correlated linearly with the extent of co-localization of PNC-27 with membrane-bound HDM-2 [17]. In addition, we found that the anti-HDM-2 antibody, but not control antibodies, blocks PNC-27 from inducing cancer cell death in a dose-dependent manner, strongly suggesting that it is the PNC-27-HDM2 membrane complexes that ultimately induce cancer cell death [22].

### 5.2. Expression of HDM-2 on the Cancer Cell Membrane Is Necessary for PNC-27-Induced Tumor Cell Necrosis

We transfected untransformed MCF-10-2A human breast epithelial cells that remain viable when treated with PNC-27, using plasmids based on the Origene Precision Shuttle Destination Vector, expressing different forms of HDM-2, including full-length HDM-2, full-length HDM-2 with a nuclear localization peptide sequence on its carboxyl terminal end that targets proteins to the cell membrane [13], and del1-109HDM-2 (missing the p53/PNC-27 binding domain) attached to the nuclear localization peptide. The plasmid used was designed by Origene, called the Precision Shuttle Destination Vector. Western blots of the membrane fractions of the two cell lines expressing the HDM-2 proteins with the membrane localization peptide showed high levels of expression in the membrane fraction. In contrast, Western blots of the membrane fractions of cells transfected with an empty vector or transfected with full-length HDM-2 without the membrane localization peptide revealed the absence of HDM-2. PNC-27 was found to colocalize with the membrane-expressed HDM-2 with the membrane localization peptide on cells that had been transfected, with the plasmid expressing this form of HDM-2 but not in cells transfected with an empty vector or with full-length HDM-2, with no membrane localization peptide [13].

Each of these transfected cell lines was incubated with PNC-27 to determine if they were killed by this peptide. Only the cells expressing full-length HDM-2 with the membrane localization peptide were killed by PNC-27. These results suggest that the interaction between PNC-27/28 with the p53 binding site (residues 1–109) of membrane-expressed HDM-2 results in tumor cell necrosis. Since cancer cells treated with either peptide have been found to have transmembrane pores (Figure 3), we surmised that the PNC-27/28-membrane-expressed HDM-2 induced pore formation. Since the isolated p53 12–26 peptide has no effect on cancer cells [13], the presence of the leader sequence evidently is vital to the process of tumor cell necrosis. We therefore sought to determine how the leader sequence would affect the interaction between PNC-27 and residues 1–109 of the HDM-2-binding site and whether complexes of PNC-27 and HDM-2 are involved in the structure of the EM-observed transmembrane pores.

### 5.3. Putative Structure for the PNC-27-1-109 HDM-2 Complex

**Figure 5:**
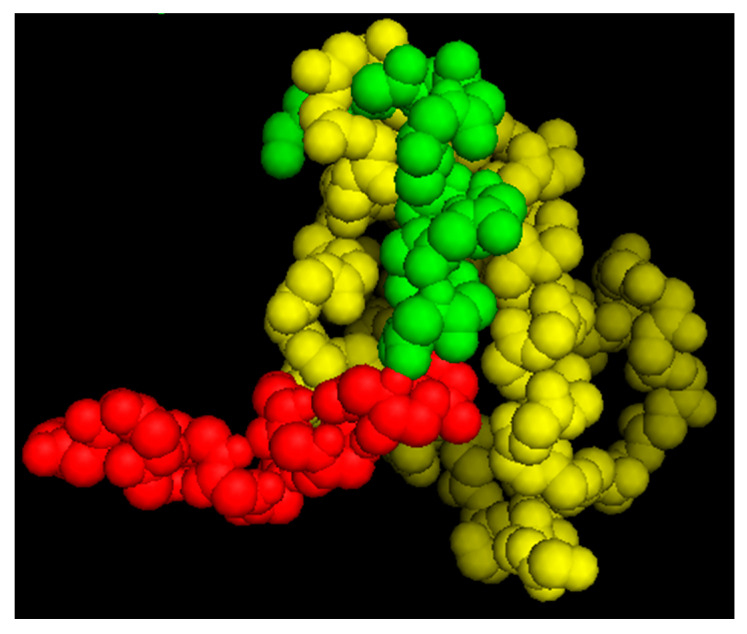
Space-filling model of the predicted structure of PNC-27 (green residues: p53 residues 12–26, red residues: leader sequence) bound to the HDM-2 p53 binding site (yellow residues) involving amino acid residues 1–109 of HDM-2, showing that the leader sequence pointed away from HDM-2 and may be involved in pore formation (Reprinted from [23]).

As noted above, we determined the solution structure of PNC-27 by 2D-NMR [13] and found that p53 residues 17–26 were superimposable [13] on the X-ray crystal structure of the 15–29 p53 peptide bound to HDM-2 residues 1–109 [12]. Using this superimposed structure of PNC-27 in the HDM-2 binding site as a starting structure for systematic energy minimizations, we obtained a lowest energy structure, as shown in Figure 5. The structure of residues 15–26 remained superimposable on the corresponding residues of the 15–29 peptide in the X-ray structure. In addition, there were critical binding residues in PNC-27 and the 15–29 peptide, including Leu 22, Trp 23, and Leu 26 that form part of the hydrophobic face of the peptide and interact closely with HDM2 residues Ile 99, Leu 54, Ile 61, and Met 62. These critical interactions were preserved in both structures (our structure for PNC-27 and the X-ray structure of the p53 15–29 peptide). In the PNC-27 final energy-minimized structure, the leader sequence projected away from the PNC-27-HDM-2 complex, making few contacts with HDM-2, suggesting that it is free to interact with other molecules in the membrane, possibly other PNC-27-HDM-2 complexes resulting in the transmembrane pores. However, in view of the absence of a structure for the whole HDM-2 protein, the orientation of the leader sequence in the PNC-27-HDM-2 complex remains to be determined.

## 6. Structure of PNC-27-Induced Transmembrane Pores

To help determine if the PNC-27-HDM-2 complex is involved in transmembrane pore formation, we performed high-resolution immune scanning electron microscopy (SEM) with high backscatter on cancer cells treated with PNC-27. In these studies, we incubated the cancer cells treated for short periods with PNC-27 and then with gold-labeled secondary antibodies in the anti-PNC-27 and anti-HDM-2 antibody system described for the colocalization experiments. Uniform gold particles of 6µ were used in the anti-PNC-27 antibody system and 15 µ for the anti-HDM-2 antibody system.

**Figure 6:**
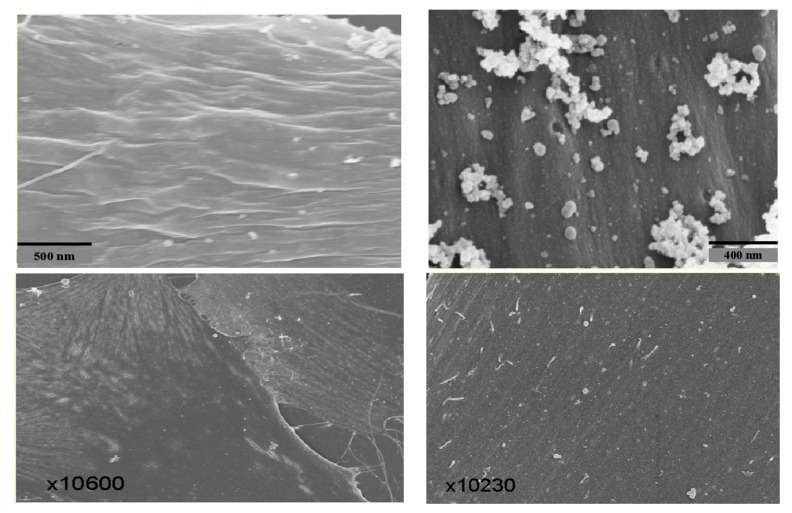
PNC-27 induced transmembrane pores in MIAPaCa-2 pancreatic cancer cells but not in normal AG13145 fibroblasts. Both cell lines were incubated for 15 min with 100 μg/mL PNC-27 for 15 min, washed, and subjected to scanning electron microscopy (SEM) with high backscatter. (**Upper panels**): left: untreated MIA-PaCa-2 cell showing smooth but ruffled membrane. Right: MIA-PaCa-2 cells treated with PNC-27, showing discrete membrane pores surrounded by stacked spheroid particles. (**Lower panels**): left: untreated AG13145 human fibroblast showing a smooth membrane. Right: AG13145 cell treated with PNC-27, again showing a smooth membrane with no pores. Adapted from [23].

Figure 6 (upper left) shows the SEM of the cell membrane of an untreated MIA-PaCa-2 pancreatic cancer cell as a ruffled surface (caused by oncogenic ras-p21). In contrast, Figure 6 (upper right) shows the membrane surface of a MIA-PaCa-2 cell that was treated with PNC-27 for 15 min. There were multiple sphere-shaped rings around holes representing transmembrane pores. Figure 6 (lower left) shows the SEM of the membrane of a typical, normal, untreated AG13145 fibroblast, and Figure 6 (lower right) shows the membrane of the fibroblasts treated with PNC-27 for 15 min. No transmembrane pores were seen. These figures indicate that PNC-27 directly induces transmembrane pores in cancer cells only.

**Figure 7:**
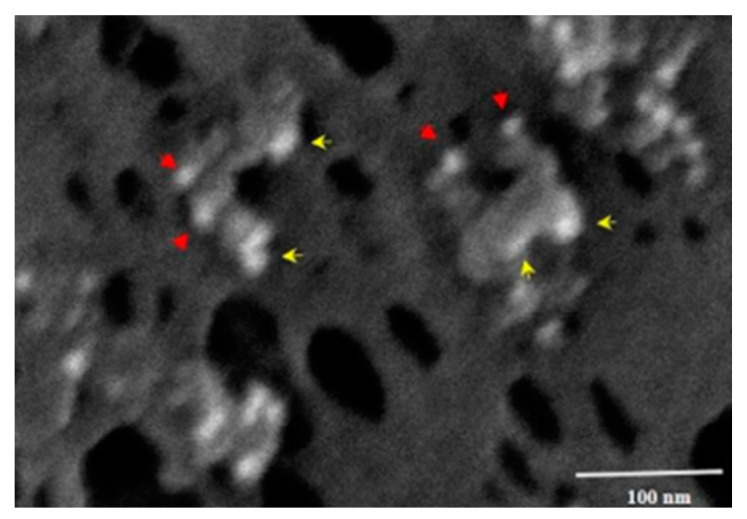
SEM showing transmembrane pores induced by PNC-27 in MIA-PaCa-2 cells were lined by dimeric complexes of gold-labeled PNC-27 (6 nm gold-labeled spheroids, labeled with red arrows) bound to gold-labeled HDM-2 (15 nm spheroids, labeled with yellow arrows). In these experiments, MIA-PaCa-2 cells were incubated with PNC-27, washed, and then incubated with the anti-PNC-27 6 nm gold-labeled antibody system and with the anti-HDM-2 15 nm gold-labeled system. Adapted from [23].

Figure 7 shows the membrane of a MIA-PaCa-2 cell treated with PNC-27 and then incubated with the two gold-labeled antibody systems. As can be seen in this figure, the transmembrane pores were formed from complexes of PNC27, labeled with the yellow arrows pointing to the 6u gold-labeled structures, with HDM-2, labeled with red arrows pointing to the 15 µ gold-labeled structures. Thus, the pores were lined with PNC-27-HDM-2 complexes, a finding that is discussed further below. These complexes appeared to occur as doublets, i.e., two sets of PNC-27–HDM-2 complexes lining a single pore. The average pore size was 37.7 nm. Thus, PNC-27 and -28 complexed with cancer-cell-membrane-bound HDM-2 directly formed transmembrane pores in cancer cells.

PNC-27-induced pore formation shows parallels with other known transmembrane-pore-inducing agents such as streptolysin O, which has been found to induce transmembrane channels by associating with membrane cholesterol, resulting in multimers of streptolysin–cholesterol complexes that line the transmembrane pores [21,24]. The formation of these complexes involved two discreet steps: a relatively temperature-independent streptolysin–cholesterol binding event followed by a strongly temperature-dependent multimer-forming process resulting in pore formation [21,24].

We found that PNC-27 induces pore formation in two similar steps [23]. Incubation of PNC-27 with a variety of cancer cells such as MIA-PaCa-2 (human pancreatic cancer) and A2058 (human melanoma) at 17 °C resulted in the absence of LDH release (no cytotoxicity), minimal cytotoxicity at 25 °C but 100 percent LDH release, and cytotoxicity at 37 °C. When these cells were incubated with PNC-27 at 17 °C and were then washed at this temperature, removing unbound PNC-27, and then were incubated at 37 °C, 100 percent cell killing occurred. The dose–response curve for the re-incubated cells was identical to that observed when the cells were incubated at 37 °C only. These findings suggest that binding of PNC-27 to membrane-bound HDM-2 is the temperature-independent step, followed by a strongly temperature-dependent co-migration event in which PNC-27–HDM-2 complexes diffuse in the cell membrane to form the transmembrane pores lined by what appear in Figure 7 to be dimers of PNC-27–HDM-2 complexes and possibly higher multimeric forms, resulting in poptosis of these cells.

### 6.1. Dependence of Pore Formation on Membrane-Associated E-Cadherin

In earlier studies [25], HDM-2 was found in metastatic breast cancer cell lines to occur in the membranes of these cells and interacted with the cell–cell adhering protein E-cadherin in the promotion of contact inhibition, inducing its ubiquitination and proteosomal degradation. In the in vivo studies on the effects of PNC-27 on human AML cells, a further investigation of the possible interactions of PNC-27 with E-cadherin were carried out [17]. (These studies indicated paradoxically that PNC-27 activated HDM-2-induced ubiquitination of E-cadherin, both in cell-free systems and in AML cells, but not in normal human stem cells.) In addition, human MV4–11 (monomyelocytic) AML cells were transfected with lentivirus-containing E-cadherin silencing (sh) RNA or control shRNA, and they were found to undergo tumor cell necrosis (termed necrobiosis), i.e., rapid release of LDH, pore formation, and consequent cell death, unlike the same cells transfected with a control vector. Interestingly, human AML blast cells treated with inactivating anti-E-cadherin antibody diminished the cytoxicity of PNC-27 to these cells, as did the proteosomal inhibitory drug bortezumib, presumably blocking the degradation of ubiquitinated E-cadherin. These results suggest the possibility that the degradation of membrane-bound E-cadherin is necessary for transmembrane pore formation and that E-cadherin blocks this process, as well as the fact that pore formation is independent of the presence of PNC-27, which acts to enhance E-cadherin degradation. However, these findings may also be explained by a blockade by these agents of PNC-27 from the interaction with its target, HDM-2.

Furthermore, our SEM studies suggest that PNC-27 is required for pore formation. As shown in Figure 7, PNC-27 forms discrete membrane complexes with HDM-2 lining the pores, clearly implicating PNC-27 directly in pore formation. In addition, if the activation of membrane-bound HDM-2 to E-cadherin degradation is the unique requirement for transmembrane pore formation, one would expect the known HDM-2-binding agents such as the p53 12–26 peptide [13] and the drug nutlin [26] to activate this process. As noted above, we found that the p53 12–26 peptide had no effect on cancer cell growth but, as shown in Figure 2, when expressed via transfected plasmid into cancer cells, interacted with intracellular HDM-2 to induce apoptosis of these cells by preventing it from binding to p53 and targeting it for ubiquitination and proteosomal degradation [13]. Nutlin, which is an HDM-2-binding drug [26], likewise induces apoptosis of cancer cells by blocking intracellular HDM-2 from binding to p53. One would expect this agent to interact with membrane-bound HDM-2 and, presumably, would activate degradation of E-cadherin, resulting in pore formation that has not been observed. There is also the known phenomenon of cancer cells [27], such as lobular breast carcinoma cells, which are membrane E-cadherin-depleted but do not exhibit pore formation and are viable [28]. Moreover, we found that PNC-27 is cytotoxic to several breast cancer cell lines (MDA-MB-157 and MDA-MB-453) that are known not to express E-cadherin [13].

### 6.2. Model for the Transmembrane Pore Formation Induced by PNC-27 and -28

**Figure 8:**
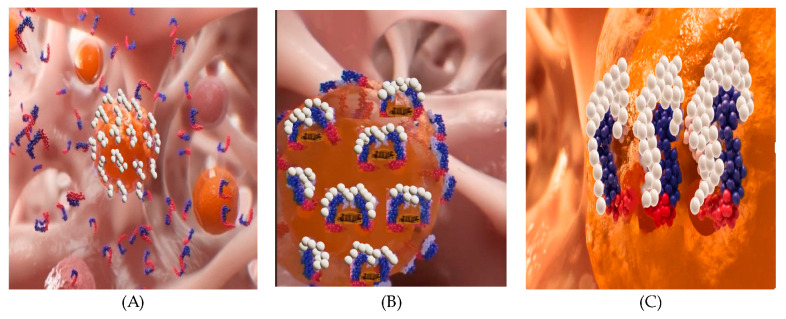
Model for PNC-27- and PNC-28-induced transmembrane pore formation in cancer cells, shown as occurring in three discrete steps: (**A**) Cancer cells expressing membrane-bound HDM-2, shown as large white molecules on the orange cancer cell surface being in contact with PNC-27, shown as the p53 12–26 sequence (blue) attached to the leader sequence (red). (**B**) PNC-27 binds to membrane-exposed HDM-2 in a temperature-independent step, represented by the complex of the blue-red PNC-27 attached to the white HDM-2 molecules. (**C**) The PNC-27-HDM-2 monomeric complexes associate by diffusion through the membrane environment in a temperature-dependent step to form dimers and possibly higher-order complexes such as to line channels through the membrane.

Figure 8 shows a model for the action of PNC-27 (and PNC-28) on cancer cells. In Figure 8A, PNC-27 shown as a helix (blue p53 residues 12–26)-loop-helix (red leader sequence) is present around the cancer cell membrane, which expresses membrane-bound HDM-2 (white structures). In Figure 8B, PNC-27 forms dimeric complexes with HDM-2. This results in multiple dimeric complexes present in the membrane, but no pore formation occurs. In Figure 8C, pairs of dimeric PNC-27-HDM-2 complexes form intramembranously, with each pair lining a transmembrane pore. It is assumed that these dimers line the pores based on the immuno-SEM structure of PNC-27-HDM-2 complexes shown in Figure 7, although this does not preclude the possibility of higher-order multimer formation.

## 7. Assessment

PNC-27 and PNC-28 peptides induce cancer cell death by a novel mechanism involving an interaction with membrane-expressed HDM-2 that appears to be unique to cancer cells since a wide variety of normal cells express either no or low levels of this protein in their membranes. Binding of these peptides to HDM-2 results in a two-step process in which the peptides bind to HDM-2 and the complexes that form associate to form transmembrane pores, resulting in an explosive release of intracellular contents (poptosis). Both peptides have been found to eradicate a solid tissue cancer and a number of human acute myelogenous leukemias in vivo, with minimal off-target effects, which is consistent with our finding that these peptides do not affect normal cells in culture.

Our results also call attention to the membranes of cancer cells as possible targets for selective killing. In a separate study [8] reviewed recently [3] and referred to in the Introduction section, a specific cell-penetrating peptide called BR2 with the sequence RAGLQFPVGRLLRRLLR selectively enters cancer cells by interacting with gangliosides in the cancer cell membrane. Using BR2 attached to an anti-ras-p21-inactivating antibody to enter colon cancer cells, this construct was found to selectively kill these cells [8]. Thus, specific membrane components of cancer cell membranes may enable selective cell killing.

Overall, the findings that PNC-27 and PNC-28 are selectively cytotoxic to cancers in vivo with minimal off-target effects due to their binding to HDM-2 expressed uniquely in cancer cell membranes suggest that these agents have strong potential in treating a variety of human cancers.

## Figures and Tables

**Table 1:** Sequences of anti-cancer peptides ^1^ and the negative control peptide ^2^.

**1. P(12)-P-L-S-S-Q-E-T-F-S-D-L-W-K-L-L**-*K-K-W-K-M-R-R-N-Q-F-W-V-K-V-Q-R-G*	**PNC-27**
**2. Q(17)-E-T-F-S-D-L-W-K-L-L(26)-** *K-K-W-K-M-R-R-N-Q-F-W-V-K-V-Q-R-G*	**PNC-28**
**3. M-P-F-S-T-G-K-R-I-M-L-G-D-** *K-K-W-K-M-R-R-N-Q-F-W-V-K-V-Q-R-G*	**PNC-29**

^1^ For the first two peptides, the numbers above the peptide sequences in bold are the amino acid sequence numbers in p53. The italicized sequence for each peptide is the leader sequence. ^2^ The bold sequence for the negative control peptide, sequence 3, was x-13 from human cytochrome P 450.

## Data Availability

Not applicable.

## References

[1] Anand U., Dey A., Chandel A.K.S., Sanyal R., Mishra A., Pandey D.K., DeFalco V., Upadhyay A., Kandimalla R., Chaudhary A. (2023). Cancer Chemotherapy and Beyond: Current Status, Drug Candidates, Associated Risks and Progress in Targeted Therapeutics. Genes Dis..

[2] Tan S., Li D., Zhu X. (2020). Cancer Immunotherpay: Pros, Cons, and Beyond. Biomed. Pharmacother..

[3] Pincus M.R., Lin B., Patel P., Gabutan E., Zohar N., Bowne W.B. (2022). Peptides That Block RAS-p21 Protein-Induced Cell Transformation. Biomedicines.

[4] Golshani G., Zhang Y. (2020). Advances in Immunotherapy for Colorectal Cancer: A Review. Ther. Adv. Gastroenterol..

[5] Ning X., Yu Y., Shao S., Deng R., Yu J., Wang X., She X., Huang D., Shen X., Duan W. (2021). The Prospect of Immunotherapy Combined with Chemotherapy in Patients with Advanced Non-Small Cell Lung Cancer: A Narrative Review. Ann. Transl. Med..

[6] Zhang K., Kong X., Li Y., Wang Z., Zhang L., Xuan L. (2022). PD-1/PD-L1 Inhibitors in Patients with Preexisting Autoimmune Diseases. Front. Pharm..

[7] Marqus S., Pirogova E., Piva T.J. (2017). Evaluation of the Use of Therapeutic Peptides for Cancer Treatment. J. Biomed. Sci..

[8] Lim K.J., Sung B.H., Shin J.R., Lee Y.W., Kim D.J., Yang K.S., Kim S.C. (2013). A Cancer Specific Cell-Penetrating Peptide, BR2, for the Efficient Delivery of an scFv into Cancer Cells. PLoS ONE.

[9] Pasquereau-Kotula E., Habault J., Kroemer G., Poyet J.-L. (2018). The Anticancer Peptide RT53 Induces Immunogenic Cell Death. PLoS ONE.

[10] Habault J., Thonnart N., Pasquereau-Kotula E., Bagot M., Bensussan A., Villoutreix B.O., Marie-Cardine A., Poyet J.-L. (2021). PAK1-Dependent Antitumor Effect of AAC-11-Derived Peptides on Sezary Syndrome Malignant CD4D T Lymphocytes. J. Investig. Dermatol..

[11] Habault J., Thonnart N., Ram-Wolff C., Bagot M., Bensussan A., Poyet J.-L., Marie-Cardine A. (2022). Validation of AAC-11-Derived Peptide Anti-Tumor Activity in a Single Graft Sézary Patient-Derived Xenograft Mouse Model. Cells.

[12] Kussie P.H., Gorina S., Marechal V., Elenbaas B., Moreau J., Levine A.J., Pavletich N.P. (1996). Structure of the MDM2 Oncoprotein Bound to the p53 Tumor Suppressor Transactivation Domain. Science.

[13] Pincus M.R., Fenelus M., Sarafraz-Yazdi E., Adler V., Bowne B., Michl J. (2011). Anti-Cancer Peptides from Ras-P21 and P53 Proteins. Curr. Pharm. Des..

[14] Thadi A., Gleeson E., Khalili M., Shaikh M.F., Goldstein E., Morano W.F., Daniels L.M., Grandhi N., Glatthorn H., Richard S.D. (2020). Anti-Cancer Tumor Cell Necrosis of Epithelial Ovarian Cancer Cell Lines Depends on High Expression of HDM-2 Protein in Their Membranes. Ann. Clin. Lab. Sci..

[15] Thadi A., Morano W.F., Khalili M., Babcock B.D., Shaikh M.F., Foster D.S., Piazza Y., Gleeson E.M., Goldstein E., Steele L. (2021). Molecular Targeting of H/MDM-2 Oncoprotein in Human Colon Cancer Cells and Stem-like Colonic Epithelial-derived Progenitor Cells. Anticancer Res..

[16] Thadi A., Lewis L., Goldstein E., Agarwal A., Khaili M., Steele L., Polyak B., Seydafkan S., Bluth M.H., Ward K.A. (2020). Targeting Membrane HDM-2 by PNC-27 Induces Necrosis in Leukemia Cells but Not in Normal Hematopoietic Cells. Anticancer Res..

[17] Wang H., Zhao D., Nyguyen L.X., Wuz H., Ling L., Dong D., Troadec E., Zhu Y., Hoang D.H., Stein A.S. (2019). Targeting Cell Membrane HDM2: A Novel Therapeutic Approach for Acute Myeloid Leukemia. Nat. Leuk..

[18] Davitt K., Babcock B.D., Fenelys M., Poon C.K., Sarkar A., Trivigno V., Zolkind P.A., Matthew S.M., Grinkina N., Orynbayeva Z. (2014). The Anti-Cancer Peptide, PNC-27, Induces Tumor Cell Necrosis of a Poorly Differentiated Non-Solid Tissue Human Leukemia Cell Line that Depends on Expression of HDM-2 in the Plasma Membrane. Ann. Clin. Lab. Sci..

[19] Bowne W.B., Sookraj K.A., Vishnevetsky M., Adler V., Yadzi E., Lou S., Koenke J., Shteyler V., Ikram K., Harding M. (2008). The Penetratin Sequence in the Anti-Cancer PNC-28 Peptide Causes Tumor Necrosis Rather than Apoptosis of Human Pancreatic Cancer Cells. Ann. Surg. Oncol..

[20] Rady I., Siddiqui I.A., Rady M., Mukhtar H. (2017). Melittin, a Major Peptide Component of Bee Venom, and Its Conjugates in Cancer Therapy. Cancer Lett..

[21] Dathe M., Wieprecht T. (1999). Structural Features of Helical Antimicrobial Peptides. Biochim. Biophys. Acta.

[22] Krzesaj P., Adler V., Feinman R.D., Miller A., Silberstein M., Yazdi E., Pincus M.R. (2024). Anti-Cancer Peptide PNC-27 Kills Cancer Cells by Unique Interactions with Plasma Membrane -Bound hdm-2 and with Mitochondrial Membranes Causing Mitochondrial Disruption. Ann. Clin. Lab. Sci..

[23] Sarafraz-Yazdi E., Mumin S., Cheung D., Fridman D., Lin B., Wong L., Rosal R., Rudolph R., Frenkel M., Thadi A. (2022). PNC-27, A Chimeric p53-Penetratin Peptide Binds to HDM-2 in a p53 Peptide-like Structure, Induces Selective Membrane-Pore Formation and Leads to Cancer Cell Lysis. Biomedicines.

[24] Farrand A.J., Hotze E.M., Sato T.K., Wade K.R., Wimley W.C., Johnson A.E., Tweten R.K. (2015). The Cholesterol-Dependent Cytolysin Membrane-Binding Interface Discriminates Lipid Environments of Cholesterol to Support Beta-Barrel Pore Insertion. J. Biol. Chem..

[25] Yang J.Y., Zong C.S., Xia W., Wei Y., Ali-Seyed M., Li Z., Broglio K., Berry D.A., Hung M.-C. (2006). MDM2 Promotes Cell Motility and Invasiveness by Regulating Ecadherin Degradation. Mol. Cell. Biol..

[26] Vassilev L.T., Vu B.T., Graves B., Carvajal D., Podlaski F., Filipovic Z., Kong N., Kammlott U., Lukacs C., Klein C. (2004). In vivo Activation of the p53 Pathway by Small-Molecule Antagonists of MDM2. Science.

[27] Taneyhill L.A., Schiffmacher A.T. (2017). Should I Stay Or Should I Go? Cadherin Function and Regulation in the Neural Crest. Genesis.

[28] McCart Reed A.E., Kalinowski L., Simpson P.T., Lakhani S.R. (2021). Invasive Lobular Carcinoma of the Breast:The Increasing Importance of This Special Subtype. Breast Cancer Res..

